# Long Non-Coding RNA MEG3 in Metal Carcinogenesis

**DOI:** 10.3390/toxics11020157

**Published:** 2023-02-07

**Authors:** Zhuo Zhang, Sophia Shi, Jingxia Li, Max Costa

**Affiliations:** Division of Environmental Medicine, Department of Medicine, New York University School of Medicine, 341 E 25 Street, New York, NY 10010, USA

**Keywords:** metals, carcinogenesis, long non-coding RNA, MEG3

## Abstract

Most transcripts from human genomes are non-coding RNAs (ncRNAs) that are not translated into proteins. ncRNAs are divided into long (lncRNAs) and small non-coding RNAs (sncRNAs). LncRNAs regulate their target genes both transcriptionally and post-transcriptionally through interactions with proteins, RNAs, and DNAs. Maternally expressed gene 3 (MEG3), a lncRNA, functions as a tumor suppressor. MEG3 regulates cell proliferation, cell cycle, apoptosis, hypoxia, autophagy, and many other processes involved in tumor development. MEG3 is downregulated in various cancer cell lines and primary human cancers. Heavy metals, such as hexavalent chromium (Cr(VI)), arsenic, nickel, and cadmium, are confirmed human carcinogens. The exposure of cells to these metals causes a variety of cancers. Among them, lung cancer is the one that can be induced by exposure to all of these metals. In vitro studies have demonstrated that the chronic exposure of normal human bronchial epithelial cells (BEAS-2B) to these metals can cause malignant cell transformation. Metal-transformed cells have the capability to cause an increase in cell proliferation, resistance to apoptosis, elevated migration and invasion, and properties of cancer stem-like cells. Studies have revealed that MEG is downregulated in Cr(VI)-transformed cells, nickel-transformed cells, and cadmium (Cd)-transformed cells. The forced expression of MEG3 reduces the migration and invasion of Cr(VI)-transformed cells through the downregulation of the neuronal precursor of developmentally downregulated protein 9 (NEDD9). MEG3 suppresses the malignant cell transformation of nickel-transformed cells. The overexpression of MEG3 decreases Bcl-xL, causing reduced apoptosis resistance in Cd-transformed cells. This paper reviews the current knowledge of lncRNA MEG3 in metal carcinogenesis.

## 1. Introduction

It is estimated that among 70–80% of the human genome transcribed, 2% is transcribed into protein-coding mRNA. Most of the human genome contains non-coding RNAs (ncRNAs) that are transcribed but not translated into proteins [1,2]. NcRNAs are divided into small (sncRNAs) and long non-coding RNAs (lncRNAs). Unlike lncRNAs, which are linear, circular RNAs (circRNAs), newly identified ncRNAs, are covalently closed RNA molecules. LncRNAs are poorly conserved RNAs of more than 200 nucleotides in length [3,4]. The biogenesis of lncRNAs is mediated through RNA polymerase II, and the transcript is capped and polyadenylated at the 3′ and 5′ ends [5,6]. LncRNAs can also be present without polyadenylation [7,8], alternative cleavage, and splicing [9], resulting in different isoforms from the same locus [10].

LncRNAs regulate their target genes transcriptionally and post-transcriptionally through interactions with proteins, RNAs, and DNAs [11] and through various mechanisms, including recruiting transcription factors to the gene promoters, binding to enhancers and the 3′-untranslated region (3′-UTRs) of genes, and competing for microRNA (miRNA) binding sites with mRNAs [4,5]. In addition, LncRNAs mediate the splicing and function of mRNA as precursors to other ncRNAs [12].

Cancer is one of the main causes of death worldwide. Cancer is driven by genetic mutations, resulting in the dysregulation of the gene regulatory networks required to maintain cellular homeostasis [6,13]. Many of these mutations occur within regions of the genome that lack protein-coding capacity, such as ncRNAs [14]. LncRNAs function as oncogenes or tumor suppressors, regulating various signaling pathways such as the cell cycle, survival, differentiation, and proliferation [15]. Some lncRNAs have been used as biomarkers for the poor prognosis of cancer in clinics. Using the TCGA database, 2,525 potential tumor-specific lncRNAs were identified by comparing gene expressions between tumors and matched normal tissues [5]. Among these tumor-specific lncRNAs, 1,103 are correlative of their expression levels with a cancer stage [5].

In this review, we summarize the general functions of lncRNA maternally expressed gene 3 (MEG3) in cancers and its involvement in metal carcinogenesis.

## 2. MEG3

Meg3 was first identified in mice as gene trap locus 2 (Gtl2) at chromosome 12. In humans, MEG3 is located at chromosome 14q32.3. The predominantly expressed isoform designated as MEG3 (isoform variant 1, GenBank NR_002766) is approximately 1.6 kb nucleotides in length. MEG3 contains multiple imprinted genes, including maternally and paternally imprinted ones, and it belongs to the Delta-like 1 *(*DLK1)-MEG3 locus [16]. The DLK-MEG3 contains two differentially methylated regions (DMRs), including intergenic DMR (IG-DMR), which is located approximately 13 kb upstream of the MEG3 transcription start site [17], and the MEG3-DMR, which overlaps with the MEG3 promoter region [16]. The hypermethylation of MEG3-IG or MEG3-DMR is correlated with reduced MEG3 transcripts, resulting in the increased expression of DLK1 and cell proliferation [18].

MEG3 is retained in the nucleus because of its nuclear retention element (NRE), a 365-nucleotide element, which is located at exons 3, 4, and 6 [19]. The alternative RNA splicing of MEG3 generates approximately 12 transcript isoforms in exons 1–4 and 8–10 [19]. Those isoforms include MEG3 (the first reported expressed sequence) and MEG3a-MEG3k. Among the isoforms, variant 1, also known as MEG3, is the predominant transcript [20]. Using the mfold program to analyze the secondary RNA folding structure revealed that all isoforms of MEG3 contain three secondary folding motifs, M1, M2, and M3 [21]. Motifs M2 and M3 are essential for p53 activation, indicating that the right folding of MEG3 is important for its biological functions [21]. MEG3 is expressed in many normal tissues, such as the adrenal gland, brain, liver, ovary, pancreas, pituitary gland, placenta, and spleen, with the highest expression in the pituitary gland and brain [21]. The expression pattern of MEG3 isoforms is tissue- and cell-type-specific [21]. For example, all 12 isoforms of MEG3 are expressed in the human fetal liver. Five isoforms, including MEG3, MEG3b, MEG3d, MEG3e, and MEG3g, are expressed in pituitary tissues [21].

### 2.1. MEG3 and Cancer

MEG3 is the first lncRNA to be identified as a tumor suppressor [21]. MEG3 is involved in a variety of cancers through the inhibition of cell growth/proliferation, migration, and invasion, and the promotion of cancer cell apoptosis. The expression of MEG3 in many primary human tumors is significantly lower than that in normal adjacent tissues, including neuroblastomas [22], lung cancers [23], liver cancers [24], gliomas [25], prostate cancers [26], and colon cancers [27]. It has been reported that loss of MEG3 was correlated with tumor grade; in meningiomas, MEG3 has been detected in four of nine grade I, one of eleven grade II, and none of seven grade III [28]. Moreover, the low expression or deletion of MEG3 was associated with advanced tumor node metastasis stage and poor prognosis in non-small-cell lung cancer (NSCLC) [29], colorectal cancer [30], gastric cancer [31], and cervical cancer [32].

The downregulation of MEG3 was also observed in various cancer cell lines. MEG3 was lost in the cells of a patient with a microdeletion in the DMR-MEG3 region, suggesting that this region is important for MEG3 gene expression [19]. The MEG3 promoter is located within the DMR-MEG3 region. Studies have demonstrated that promoter hypermethylation and gene deletion resulted in the loss of MEG3 in tumor tissues and cancer cell lines [19]. The results from bisulfite sequencing analysis showed that the methylation in the MEG3 promoter was dramatically increased in nonfunctioning adenomas compared to normal pituitary [33]. Hypermethylation in the MEG3 promoter was also found in meningiomas and neuroblastoma cell lines [22,34]. The deletion of the cAMP response element (CRE) at the transcription factor-binding site of the MEG3 promoter dramatically decreased the promoter activity of MEG3 [34]. The hypermethylation of the MEG3 promoter was also detected in retinoblastoma tissues and was correlated with the low expression of MEG3 and the poor survival of retinoblastoma patients [35]. Reduced MEG3 expression by the hypermethylation of the MEG3 promoter increased β-catenin expression, promoting proliferation and inhibiting apoptosis in retinoblastoma cells [35]. The hypermethylation of the MEG3 promoter was also observed in cervical cancer [32] and NSCLC [36]. In gastric cancer, treatment with 5-Aza-CdR, a demethylating agent, elevated levels of both MEG3 and p53, causing the inhibition of cellular proliferation [37]. In glioma cells, 5-Aza-CdR increased MEG3 expression, suppressing cell proliferation via the downregulation of the Wnt/β-catenin pathway [28]. These studies suggested that in tumor tissues and cancer cells, the hypermethylation of the MEG3 promoter caused its downregulation and that the inhibition of methylation increased MEG3 levels.

### 2.2. Mechanisms of MEG3 in Cancer Initiation, Progression, and Development

The mechanisms of MEG3 inhibiting carcinogenesis have been intensively studied recently. MEG3 functions as a tumor suppressor through the activation of the p53 pathway [38,39]. However, MEG3 was able to inhibit cell proliferation in the absence of p53 [40], indicating that MEG3 acts as a tumor suppressor both in p53-dependent and -independent pathways [40]. MEG3 inhibited oncogenic C-Myc and β-catenin activity in liver cancer [40]. MEG3 caused G0/G1 arrest in prostate cancer cells [41] and G2/M arrest in cervical cancer cells [42]. The restoration of MEG3 expression suppressed tumor growth [43] and induced apoptosis in several human cancer cell lines, including lung cancer A549 cells [44], tongue squamous cell carcinoma cell lines SCC-15 and CAL27 [45], and gastric cancer cell lines SGC7901, AGS, MGC803, MKN45, and BGC823 [37]. The major pathways of MEG3 in the regulation of cancer cell proliferation, migration, and invasion are summarized in the following subsections.

#### 2.2.1. p53 Pathway

Tumor suppressor p53 is associated with both cancer initiation and progression. The overexpression of MEG3 markedly increased the p53 protein level and stimulated p53-dependent transcription from a p53-responsive promoter [46]. Another study indicated that the overexpression of MEG3 increased both mRNA and protein levels of p53 in MCF-7 and ZR75-1 breast cancer cells [47]. Deletion mutations of MEG3 failed to stimulate p53 transcriptional activity, suggesting that the full length of MEG3 was required for p53 transcription [47]. An additional study demonstrated that the DNA binding domain (DBD) of p53 was responsible for the direct interaction between p53 and MEG3 and the M3 domain of MEG3 was critical for this direct interaction [48]. The binding of MEG3 to the p53 promoter caused the accumulation of p53 protein, inducing p53-target genes [21]. The knockdown of p53 markedly reduced the influences of MEG3 overexpression on apoptosis, migration, and invasion, suggesting that p53 was involved in apoptosis, migration, and invasion regulated by MEG3 in breast cancer cells [47]. The results from a microarray analysis showed that out of 287 target genes of p53, thirty-four were regulated by MEG3 [48]. Among these thirty-four genes, twenty-one were upregulated and thirteen were downregulated [48]. These genes regulate cell death, apoptosis, cell proliferation, and cell cycle. MEG3 may function as a coactivator of p53 in the activation of its transcriptional activity. Because p53 tetramerization is important in the regulation of p53 activity [49], it is suggested that MEG3 may activate p53 through promoting p53 tetramerization.

The capacity of different MEG3 isoforms to regulate p53 and thus cell proliferation is dependent on the degree of the methylation of the promoter [47,50]. Factors causing the splicing of MEG3 affect the capacity of MEG3 RNA to regulate p53 and consequently apoptosis, cell proliferation, and cell cycle. It was reported that all MEG3 isoforms were able to stimulate p53-mediated transactivation and suppress DNA synthesis in colon cancer cells [27]. While the stimulation of p53-mediated transactivation varied between the different MEG3 isoforms, the suppression of DNA synthesis by each MEG3 isoform was similar [21]. Similarly, MEG3 inhibited the cell proliferation of HCT116 and DLD-1 cells through p53 activation [30]. MEG3 induced growth differentiation factor 15 (GDF15), an inhibitor of cell proliferation, the expression of which is dependent on p53, resulting in the suppression of cell proliferation in HCT116 cells [46]. MEG3 was lost or reduced in the majority of hepatocellular carcinoma (HCC) tissues compared to adjacent normal tissues. The ectopic expression of MEG3 in hepatoma cells significantly inhibited proliferation and induced apoptosis [48]. MEG3 was able to upregulate GADD45A, a p53-responsive stress protein, playing an important role in suppressing cell proliferation and promoting apoptosis [48]. Transforming growth factor α (TGFα), a member of the epidermal growth factor (EGF) family and a direct target gene of p53, which activates signaling pathways involved in cell proliferation, differentiation, and development, was also upregulated by MEG3 [48]. In lung cancer SPC-A1 cells, the overexpression of MEG3 stimulated the protein levels of p53, leading to the suppression of cell growth in vitro and tumor growth in vivo [51].

#### 2.2.2. MDM2 Pathway

Mouse double minute 2 (MDM2), a nuclear E3 ubiquitin ligase, mediates protein ubiquitination, which can cause p53 degradation. MEG3 suppressed MDM2 expression and the downregulation of the MDM2 protein was due to p53 activation by MEG3 [46]. Increased MEG3 expression upregulated p53 by suppressing MDM2 in vivo [21]. In esophageal cancer mouse tissue, both forkhead box P3 (FOXP3), a protein involved in immune system response, and MDM2 were upregulated, while Meg3, miR-149-3p, and p53 were all decreased [52]. Further study indicated that Meg3 decreased the expression of the FOXP3 and upregulated miR-149-3p through MDM2-mediated p53 in esophageal cancer cells AKR [52]. The elevation of Meg3 suppressed regulatory T-cell differentiation and immune escape by suppressing FOXP3 or increasing p53 in mice with esophageal cancer [52]. In colon carcinoma cells and osteosarcoma cells, MEG3 recruited polycomb repressive complex 2 (PRC2), a chromatin-modifying enzyme, which catalyzes the methylation of histone H3 at lysine 27 (H3K27me1/2/3) on the MDM2 promoter, causing MDM2 downregulation and p53 activation [46]. MDM2 levels were reduced in HCT116 cells transfected with MEG3, indicating that MDM2 suppression by MEG3 contributes at least in part to p53 accumulation [46].

#### 2.2.3. Rb Pathway

Retinoblastoma protein (Rb) is another important tumor suppressor. MEG3 regulates cell proliferation through the RB transcriptional corepressor 1 pathway [53]. The results from a microarray analysis of mouse embryonic fibroblasts (MEFs) isolated from mice with the genetic deletion of all three Rb family members showed a significant silencing of Meg3 expression compared to MEFs from wild-type mice [44]. MEG3 levels were also suppressed in A549 lung cancer cells compared with normal human bronchial epithelial (NHBE) cells, and the re-expression of MEG3 led to a decrease in cell proliferation and an elevation in apoptosis [44]. The activation of pRb by the treatment of lung cancer A549 and SK-MES-1 cells with palbociclib, a CDK4/6 inhibitor, increased the expression of MEG3, while the knockdown of pRb/p107 attenuated MEG3 gene expression [44]. The results from an analysis of the TCGA database revealed that reduced MEG3 gene expression in human lung cancers disrupted the Rb pathway [44]. Rb was induced by MEG3 expression through the downregulation of DNA methyltransferase 1 (DNMT1), resulting in a decrease in MEG3 methylation [44]. The results from methylation-specific PCR revealed that the activation of Rb by the treatment of tongue squamous cell carcinoma (TSCC) cells with palbociclib increased unmethylated MEG3. Thus, MEG3 increased its expression and inhibited the cell proliferation [45]. It is noted that both miR-29 and miR-26a elevated MEG3 expression via repressing DNMT1 and DNMT3b [45,54].

#### 2.2.4. Wnt/β-Catenin Pathway

β-catenin, the key member of the Wnt signaling pathway, plays a key role in the regulation of cell proliferation, differentiation, apoptosis, and migration and invasion. In humans, β-catenin was mutated in 44% of HCC [55]. The treatment of retinoblastoma cells with 5-Aza-CdR caused the demethylation of the MEG3 promoter, resulting in the upregulation of MEG3 and further inactivation of the Wnt/β-catenin pathway, leading to the inhibition of cell growth [35]. A recent study indicated that MEG3 promoted β-catenin degradation via GSK-3β, which in turn inactivated the Wnt pathway and ultimately inhibited the invasion and metastasis of retinoblastoma cells [56]. Further study indicated that MEG3 promoted the binding of β-catenin to GSK-3β, inducing the phosphorylation, ubiquitination, and degradation of β-catenin [56]. The expression levels of β-catenin from metastasis retinoblastoma tissues were dramatically higher than in those in primary retinoblastoma tissues and in the normal retinal tissues [56]. Pearson correlation analysis revealed that MEG3 was negatively correlated with β-catenin in those three types of tissues [56]. A study revealed that in the colon and hepatocellular carcinoma cells there is an RNA-binding region on β-catenin, which can bind to lncRNA to regulate the protein function [57].

GSK-3β regulates the Wnt pathway by phosphorylating β-catenin at several phosphorylation sites, including Ser33, Ser37, and Thr41. The inhibition of GSK-3β by its shRNA significantly reduced the role of MEG3 in regulating β-catenin and in promoting the invasion of retinoblastoma cells [56], suggesting that the GSK-3β protein is an important intermediator for the MEG3 functions. In lung adenocarcinoma cells, lncRNA bound to GSK-3β, thereby regulating the function of GSK-3β [58]. LncRNA linc00261 bound to both GSK-3β and Slug protein, promoting the degradation of the Slug protein through GSK-3β, thus inhibiting the invasion and metastasis of gastric cancer cells [59]. It is speculated that MEG3 regulates the proliferation, invasion, and migration of cancer cells through its binding to the GSK-3β protein.

#### 2.2.5. MicroRNAs

MicroRNAs (miRNAs), small non-coding RNAs, regulate their target gene expression by complementary base pairing with the 3′-UTRs of mRNAs [60]. The aberrant expression of miRNAs is a hallmark of human cancer [61]. Some miRNAs are located in the intron of MEG3 [62]. The miRNAs derived from the MEG3 transcript are controlled by the MEG3 promoter [63]. It has been reported that twenty-nine miRNAs were associated with disease outcomes in advanced ovarian cancer patients and eleven of them were in the DLK1-MEG3 cluster [64]. Ten of these miRNAs, including miR127, miR-154, miR-299-3p, miR-376c, miR-377, miR-381, miR-382, miR-388, miR-409-3p, and miR-433, were associated with overall survival [64]. MEG3 regulated cancer development by suppressing miRNAs in several types of cancer [65]. MEG3 functioned as a molecular inhibitor of miR-7-5p and miR-3163 to suppress NSNLC cell growth [66,67]. MEG3 decreased the cell migration and invasion of NSCLC PC9 and H1299 cells via suppressing miR-21-5p, resulting in elevated PTEN expression levels, partially via the PI3k/Akt pathway [68]. The restored expression of MEG3 in breast and ovarian cancer cells suppressed the PI3k/Akt pathway with the concomitant elevation of PTEN expression [69,70]. MEG3 negatively regulated cell proliferation by suppressing miR-19a, which in turn targeted PTEN in glioma cells [71]. Further study showed that MEG3 inhibited the epithelial–mesenchymal transition (EMT) process by upregulating E-cadherin, an epithelial marker, and by downregulating N-cadherin and Vimentin, mesenchymal markers, in PC9 and H1299 cells [68]. The results from our recent study showed that MEG3 decreased developmentally downregulated protein 9 (NEDD9) and the knockdown of NEDD9 inhibited migration and invasion in human lung adenocarcinoma A549 cells [72]. It has also been reported that in glioma cells, MEG3 reduced miR-96-5p by direct binding, leading to the suppression of cell proliferation, migration, and invasion [25].

#### 2.2.6. Other Pathways Involved in Cell Proliferation, Angiogenesis, and Tumorigenesis

MEG3 can suppress cell proliferation and growth in the absence of p53 [46]. One of the mechanisms by which MEG3 exerts its function is through the cyclic AMP (cAMP) response element (CRE) [34]. The hypermethylation of the MEG3 promoter suppressed the binding of CRE to the cAMP response element binding protein (CREB), preventing the transcription initiation of MEG3 [73]. cAMP elevated MEG3 gene expression through the CRE binding site, inhibiting cell proliferation and growth [34].

The PI3k/Akt pathway is crucial for cell proliferation [74]. It has been reported that MEG3 suppressed the PI3k/Akt pathway in hepatocellular carcinoma SMMC-7721 and BEL-7402 cells, resulting in the inhibition of cell proliferation and invasion [75]. MEG3 competitively bound to miR-93, causing the inactivation of the PI3k/Akt/mTOR pathway, and ultimately the suppression of cell proliferation and autophagy and the induction of apoptosis in bladder cancer cells [76]. Similarly, MEG3 overexpression ameliorated the inhibition of miR-1297 on PTEN, reducing Akt activation, thus decreasing the cell growth of human testicular germ cell tumor (TGCT) NCCIT cells [77].

MEG3 was downregulated in breast cancer tissues and cell lines MCF-7 and MDA-MB-231 [69]. The overexpression of MEG3 markedly inhibited expressions of angiogenesis-related genes, including vascular endothelial growth factor A (VEGFA), placental growth factor (PGF), basic fibroblast growth factor (bFGF), transforming growth factor β1 (TGF-β1), and matrix metallopeptidase 9 (MMP-9) [69]. Conditioned medium aliquoted from breast cancer cells with MEG3 overexpression significantly reduced the capillary tube formation of endothelial cells [69]. Moreover, the overexpression of MEG3 in breast cancer cells inhibited tumorigenesis and angiogenesis in the nude mice xenograft model [69]. The results from quantitative RT-PCR revealed that the VEGF signaling pathway, including VEGFA and VEGFR1, and neuropilin 1 (NRP1), which binds to VEGFA to function as a co-receptor for VEGF2 during angiogenesis, were activated in the embryos from Meg3-null mice compared to those from wild-type ones [78].

The mechanisms of MEG3 in cancer development are outlined in Table 1.

## 3. MEG3 in Carcinogenesis of Heavy Metals

Most heavy metals are toxic and carcinogenic to humans. Heavy metals are widely utilized in various industrial and agricultural products, such as paints, batteries, pigments, electronic waste, and insecticides/pesticides. Contaminated heavy metals in the environment flow into soil, lake, river, and ocean through rain and groundwater, where the metals accumulate via the circulating bio-system, resulting in high concentrations in humans [79]. Chromium (Cr(VI)), arsenic (As), nickel (Ni), and cadmium (Cd) are listed as Group 1 human carcinogens by the International Agency for Research on Cancer (IARC). Studies have indicated that exposure to these metals disrupts cellular signaling pathways, such as damaged repair processes, reduced gene expression of tumor suppressors, and aberrant metabolism, leading to carcinogenesis [79].

### 3.1. MEG3 in Cr(VI) Carcinogenesis

Hexavalent chromium (Cr(VI)) compounds are widely utilized in multiple industrial activities such as wood preservation, tanning, paint production, chemical production, and electroplating. The contamination of soils and groundwater caused by the use of Cr(VI) in these industrial and agricultural practices has brought worldwide concerns about the adverse health effects on humans. The Environmental Protection Agency (EPA) and IARC have classified Cr(VI) as a confirmed human carcinogen for lung cancer. It has been reported that the chronic exposure of human bronchial epithelial cells to low doses of Cr(VI) caused malignant cell transformation [80]. These transformed cells displayed rapid cell growth [81], resistance to apoptosis [82], increased angiogenesis [83], and tumor growth in xenograft animals [84].

The mechanisms of Cr(VI)-induced carcinogenesis have been extensively studied. However, the role of long non-coding RNA, such as MEG3, in Cr(VI) carcinogenesis has not been well investigated. The results from Arraystar microarray and bioinformatic analysis showed that the short-term exposure of human bronchial epithelial 16HBE cells to Cr(VI) caused 1,868 lncRNAs upregulation and 2,203 lncRNAs downregulation [85]. MEG3 was not listed in the top 10 downregulated genes [85]. Our recent study has observed that the MEG3 expression level was reduced in BEAS-2B cells exposed to Cr(VI) and that MEG3 was lost in Cr(VI)-transformed cells [72]. The ectopic expression of MEG3 decreased the migration and invasion of Cr(VI)-transformed cells [72]. While miR145-5p was upregulated in Cr(VI)-transformed cells, the ectopic expression of MEG3 decreased miR-145-5p levels and NEDD9 protein levels [72]. The levels of miR-145-3p were increased in the plasma of workers occupationally exposed to Cr(VI) compared to those without exposure [86]. β-catenin was activated in Cr(VI)-transformed cells, the overexpression of MEG3 decreased the activation of β-catenin [72]. Further study has found that MEG3 bound to miR-145 and the mutation of MEG3 at the binding site for miR-145 was unable to alter NEDD9 expression or decrease the invasion and migration of Cr(VI)-transformed cells [72]. Interestingly, the knockdown of MEG3 in normal BEAS-2B cells promoted migration and invasion [72]. The study indicated that in Cr(VI)-transformed cells, the loss of MEG3 increased NEDD9 expression, resulting in the activation of β-catenin and the further upregulation of EMT, promoting migration and invasion [72].

### 3.2. MEG3 in Arsenic Carinogenesis

Arsenic compounds are widely used as pesticides, fungicides, herbicides, wood preservatives, and paints. The contamination of drinking water due to agricultural activities is the major exposure route for humans to arsenic compounds. Inorganic forms of arsenite and arsenate compounds have been classified as confirmed human carcinogens of the bladder, lung, and skin by EPA and IARC.

The mechanisms of arsenic carcinogenesis have been the most studied among all heavy metals that have been classified as human carcinogens, including Cr(VI), nickel, and cadmium. However, very few studies on MEG3 in arsenic carcinogenesis are available. It has been reported that paternal non-occupational exposure to environmental arsenic elevated DNA methylation levels of MEG3 in the sperm of 353 male subjects [87]. The results from a case-control study revealed that MEG3 levels in peripheral blood lymphocytes were positively correlated with the levels of inorganic arsenic (iAs), monomethylarsonic acid (MMA), and dimethylarsinic acid (DMA) in the urine of workers exposed to arsenic [88]. It has also been reported that MEG3 levels in peripheral blood were reduced in workers exposed to arsenic occupationally, while the arsenic concentrations were increased in the urine [89].

### 3.3. MEG3 in Nickel Carcinogenesis

Nickel compounds were classified as Group 1 human carcinogens by IARC in 1990 [90]. The carcinogenic effects of nickel include oxidative stress, DNA damage, inflammation, and epigenetic and genetic changes [91]. Hypoxia-inducible factor-1 (HIF-1), a transcription factor, plays an important role in the regulation of angiogenesis [92]. HIF-1α, a regulatory subunit of HIF-1, is responsible for the transcriptional function of HIF-1. Our previous study has found that nickel activated the hypoxia-inducible pathway, which is important in the nickel-induced carcinogenic process [93]. It has also been reported that nickel exposure caused HIF-1α protein accumulation, leading to malignant cell transformation [94].

Although the mechanisms of nickel carcinogenesis have been well investigated, the role of lncRNA in nickel-induced carcinogenesis has yet to be extensively studied. It has been reported that the exposure of the cells to nickel caused the downregulation of MEG3, leading to malignant cell transformation [95]. While the knockdown of MEG3 facilitated the malignant cell transformation by nickel, the overexpression of MEG3 reduced the malignant cell transformation induced by chronic exposure to nickel in BEAS-2B cells [95]. Lung squamous cell carcinomas are the major type of lung cancer induced by occupational exposure to nickel [96]. MEG3 was downregulated in the tumor tissues from human lung squamous cell carcinoma compared to those from adjacent normal lung tissues [95]. Chronic exposure of the cells to nickel downregulated MEG3, causing the transcriptional inhibition of the c-Jun-mediated PH domain and leucine-rich repeat protein phosphatase 1 (PHLPP1) and the upregulation of HIF-1α protein, leading to malignant cell transformation [95]. Further study suggested that the downregulation of MEG3 was due to the hypermethylation of the promoter through elevated DNA methyltransferase 3 beta (DNMT3b) induced by nickel. The reduced interaction of MEG3 with c-Jun inhibited PHLPP1 transcription, resulting in the upregulation of HIF-1α protein through the activation of the Akt/p70S6k/S6 pathway [95].

MEG3 was downregulated in the pulmonary fibrosis tissues of rats induced by nickel oxide nanoparticles (NiO NPs) [97]. Autophagy proteins Beclin 1 and LC3B were both downregulated and p62 was upregulated in the lung fibrosis tissues [97]. The overexpression of MEG3 was able to restore levels of Beclin1 and LC3B reduced by NiO NPs in A549 cells [97]. The Hedgehog pathway plays an important role in cell proliferation. The overexpression of MEG3 inhibited the Hedgehog pathway activated by NiO NPs exposure [97]. While the exposure of NiO NPs to rats activated the p38 MAPK and induced inflammatory responses, the overexpression of MEG3 suppressed the p38 MAPK, resulting in reduced levels of inflammatory cytokines, including IL-6, IL-8, and TNF-α [98].

### 3.4. MEG3 in Cadmium Carcinogenesis

Adverse health effect caused by exposure to cadmium (Cd) compounds is a major public health concern. Cd(II) is the common Cd oxidation state. Studies have demonstrated that Cd(II) exposure causes lung cancer and cancers of other organs [99,100,101,102,103,104,105,106,107]. IARC has classified Cd(II) as a human Group 1 carcinogen [100].

Although the mechanism of Cd(II)-induced carcinogenesis remains largely unknown, recent studies have indicated that MEG3 plays a key role in malignant cell transformation induced by Cd(II) exposure [108]. Chronic exposure of BEAS-2B cells to a low dose of Cd(II) induced malignant cell transformations and generated cancer stem cell (CSC)-like cells [108]. MEG3 level was markedly reduced in Cd(II)-transformed cells. The p21 level was reduced and the levels of Rb phosphorylation and Bcl-xL protein were increased in those Cd(II)-transformed cells [108]. The overexpression of MEG3 elevated the p21 level and decreased levels of both Rb phosphorylation and Bcl-xL, resulting in reduced apoptosis resistance of Cd(II)-transformed cells [108].

One function of the MEG3-DMR is to maintain activity in the MEG3-IG region. The hypermethylation of MEG3-DMR induced by Cd(II) altered the expression of various genes in this imprinted domain [17]. The results from a study of 287 pairs of infants–mothers showed significantly increased levels of prenatal Cd and MEG3-DMR hypermethylation in cord blood, and the associations were strongest in those born to African American women compared with those born to White women or Hispanic women, indicating that prenatal Cd exposure is associated with the aberrant methylation of the MEG3-DMR at birth [17]. Thus, it is speculated that the reductions in MEG3 transcripts by Cd cause the loss of MEG3 tumor suppression function, resulting in increased susceptibility to cancer.

The role of MEG3 in metal-induced carcinogenesis is summarized in Table 2.

## 4. Conclusions and Future Perspective

**Figure 1:**
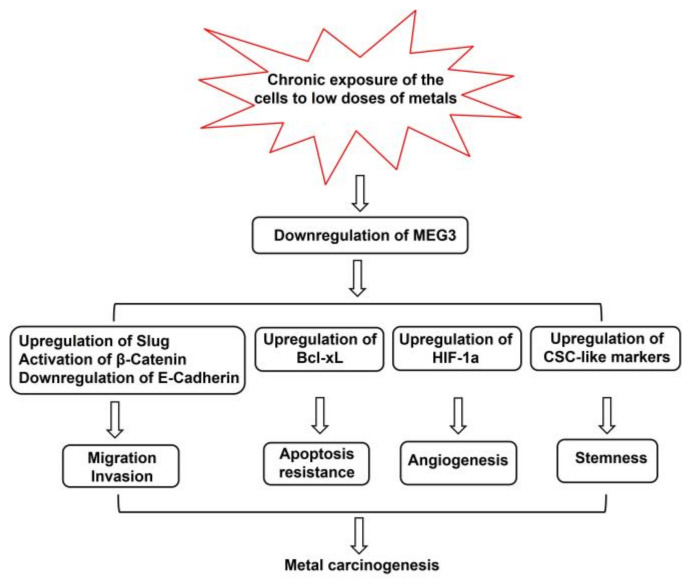
Representative mechanisms of MEG3 in metal carcinogenesis. Chronic exposure of the cells to low doses of metal reduces MEG3 gene expression. The downregulation of MEG3 causes (1) the activation of β-Catenin and upregulation of EMT, resulting in increased migration and invasion; (2) the upregulation of Bcl-xL; (3) an increase in HIF-1α; and (4) the upregulation of cancer stem cell-like markers, contributing to carcinogenesis induced by metals.

Among 70–80% of the human genome transcribed, 2% of the genome encodes protein sequences. The majority produce lncRNA. LncRNA interacts with miRNA, mRNA, and DNA, and is involved in many processes including transcriptional and translational regulation and cell development. LncRNA MEG3 acts as a tumor suppressor to increase the expression of p53 and activate p53 downstream targets, resulting in the inhibition of cancer cell proliferation and growth. Heavy metals, such as Cr(VI), arsenic, nickel, and cadmium, are confirmed human carcinogens. The mechanisms of carcinogenesis induced by these metals have been intensively studied. However, the role of MEG3 in the process of carcinogenesis by metals remains to be investigated. Several in vitro studies have demonstrated that MEG3 was downregulated in the malignantly transformed cells induced by chronic exposure to those metals. Epidemiological studies have also indicated that exposure to arsenic and cadmium reduced MEG3 gene expression in blood. However, the molecular mechanisms of MEG3 in the regulation of cell transformation by metals are largely unknown. Figure 1 shows the representative mechanisms of MEG3 in metal carcinogenesis. Since metal-transformed cells exhibit increased migration and invasion, the role of MEG3 in the aggressiveness of those transformed cells should be one of the future research directions in the field of metal toxicology. In vivo studies using MEG3 knockout animals to elucidate the importance of MEG3 in tumor development induced by metals will certainly provide valuable evidence for further understanding the mechanisms of metal carcinogenesis, including cancer prevention and intervention. Human studies on the possible correlation of metal exposure to MEG3 will also help with understanding the mechanisms of MEG3 in metal carcinogenesis.

## Figures and Tables

**Table 1:** The mechanisms of MEG3 in cancer development. MEG3 is involved in multiple processes of cancer development, such as cell proliferation, cell cycle, apoptosis, autophagy, angiogenesis, migration and invasion, tumor growth, and metastasis. The inhibitory effects of MEG3 on cancer are involved in various pathways, including p53, MDM2, Rb, Wnt/β-catenin, microRNA, PI3K/Akt, VEGF, and many others.

Pathways	Function Roles	Mechanisms	References
p53 pathway	Cell proliferation, cell cycle, and apoptosis	Stimulation of p53 transcription and stabilization of p53 protein	[21,30,46,47,48]
MDM2 pathway	Cell proliferation, cell cycle, and apoptosis	Downregulation of MDM2 protein	[21,46,52]
Rb pathway	Cell proliferation, cell cycle, and apoptosis	Rb phosphorylation	[44]
Wnt/β-Catenin pathway	Cell growth, migration, invasion, and metastasis	Phosphorylation, ubiquitination, and degradation of β-Catenin; activation of G3K-3β	[35,56,57]
microRNA	Cell proliferation, apoptosis, migration, invasion, and metastasis	Suppression of microRNAs, including miR-7-5p, miR-3163, miR-21-5p, miR-19a, and miR-96-5p	[25,66,67,68,71]
VEGF pathway	Angiogenesis and tumorigenesis	Inhibition of angiogenesis-related gene expression, such as VEGFA, PGF, bFGF, TGF-β1, and MMP-9	[69,78]
PI3k/Akt pathway	Cell proliferation, cell growth, autophagy, apoptosis, and tumor growth	Inactivation of PI3k/Akt	[75,76,77]

**Table 2:** The role of MEG3 in metal-induced carcinogenesis. Exposure to chromium (Cr(VI)), arsenic (As), nickel (Ni), or cadmium (Cd) downregulated MEG3, disrupting multiple cellular signaling pathways, leading to malignant cell transformation, inflammation, and enhanced migration and invasion. BEAS-2B: human bronchial epithelial cells. BLF: bronchoalveolar lavage fluid.

Metals	MEG3 Levels	Tissues or Cells	Target Genes	Hallmarks of Cancer	References
Cr(VI)	Downregulation	BEAS-2B cells	NEDD9, β-catenin, and EMT	Migration and invasion	[72]
As	Downregulation	Human sperms	DNA methylation of MEG3		[87]
	Downregulation	Human peripheral blood lymphocytes			[88,89]
Ni	Downregulation	BEAS-2B cells	Akt/p70S6k/S6 signaling	Cell transformation	[95]
	Downregulation	A549 cells and BLF from rats	Inflammatory cytokines and p38 MAPK	Inflammation of the lungs	[98]
Cd	Downregulation	BEAS-2B cells	p21, Rb, and Bcl-xL	Cell transformation	[108]
	Downregulation	Human cord blood	Hypermethylation of MEG3		[17]

## Data Availability

Not applicable.
